# Joint eQTL assessment of whole blood and dura mater tissue from individuals with Chiari type I malformation

**DOI:** 10.1186/s12864-014-1211-8

**Published:** 2015-01-22

**Authors:** Eric F Lock, Karen L Soldano, Melanie E Garrett, Heidi Cope, Christina A Markunas, Herbert Fuchs, Gerald Grant, David B Dunson, Simon G Gregory, Allison E Ashley-Koch

**Affiliations:** Department of Medicine, Duke University Medical Center, Durham, NC USA; Department of Statistical Science, Duke University, Durham, NC USA; Duke Center for Human Disease Modeling, Duke University Medical Center, Durham, NC USA; Division of Neurosurgery, Department of Surgery, Duke University Medical Center, Durham, NC USA; Department of Neurosurgery, Stanford University/Lucile Packard Children’s Hospital, Stanford, CA USA; Duke Molecular Physiology Institute, Duke University Medical Center, Durham, NC USA

**Keywords:** eQTL analysis, Multi-tissue integration, Chiari Type I Malformation, Dura mater, Whole genome expression

## Abstract

**Background:**

Expression quantitative trait loci (eQTL) play an important role in the regulation of gene expression. Gene expression levels and eQTLs are expected to vary from tissue to tissue, and therefore multi-tissue analyses are necessary to fully understand complex genetic conditions in humans. Dura mater tissue likely interacts with cranial bone growth and thus may play a role in the etiology of Chiari Type I Malformation (CMI) and related conditions, but it is often inaccessible and its gene expression has not been well studied. A genetic basis to CMI has been established; however, the specific genetic risk factors are not well characterized.

**Results:**

We present an assessment of eQTLs for whole blood and dura mater tissue from individuals with CMI. A joint-tissue analysis identified 239 eQTLs in either dura or blood, with 79% of these eQTLs shared by both tissues. Several identified eQTLs were novel and these implicate genes involved in bone development (IPO8, XYLT1, and PRKAR1A), and ribosomal pathways related to marrow and bone dysfunction, as potential candidates in the development of CMI.

**Conclusions:**

Despite strong overall heterogeneity in expression levels between blood and dura, the majority of cis-eQTLs are shared by both tissues. The power to detect shared eQTLs was improved by using an integrative statistical approach. The identified tissue-specific and shared eQTLs provide new insight into the genetic basis for CMI and related conditions.

**Electronic supplementary material:**

The online version of this article (doi:10.1186/s12864-014-1211-8) contains supplementary material, which is available to authorized users.

## Background

*Expression quantitative trait loci* (eQTLs) are genetic polymorphisms that affect the expression level of a gene. A variety of methods are commonly used to detect eQTLs in individual tissues [1-3]. The identification of eQTLs is important for dissection of human disease, by providing hypotheses for how genetic alterations translate to individual differences in biological function and risk for disease.

Gene expression levels are known to vary widely between different types of tissue. Consequently, the result of gene expression analysis often depends strongly on the type of tissue examined for any given experiment, and this too is applicable to the identification of eQTLs. The study of tissue-by-tissue variation is an ongoing and dynamic area of research. In particular, the *Genotype-Tissue Expression* (GTEx) project [4] is a large-scale collaborative effort to catalogue gene expression variation and genetic association with expression among several tissue types. The GTEx database now includes expression measurements and candidate eQTLs for over 20 different types of tissue. From a clinical perspective, it would be helpful to identify potential commonalities between gene expression profiles in accessible tissue (such as blood) versus more inaccessible tissue (brain, dura mater, cerebrospinal fluid) as this information could lead to the development of biomarkers for human diseases.

Despite strong tissue-to-tissue variability in gene expression, results from the GTEx project suggest that eQTLs are often, but not always, shared across multiple tissues. Therefore, when expression levels for multiple tissues are available, integrative methods that detect eQTLs across all tissues simultaneously are preferable to simply analyzing each tissue separately. Recent methods [5,6] allow for the borrowing of information across tissue types for more accurate detection of eQTLs. In this study, we present tissue-by-tissue analysis of eQTLs separately for blood and dura mater tissue, and a joint analysis across the two tissues simultaneously. We compare these two approaches to determine if the gain in statistical power from the joint analysis reveals similar or different eQTLs between the tissues.

This article describes the detection of eQTLs for both blood and dura mater tissue for 43 individuals with *Chiari type 1 malformation* (CMI). CMI is characterized by herniation of the cerebellar tonsils below the foramen magnum (base of the skull) and is estimated to affect 1% of the United States population [7]. CMI is a heterogeneous condition as the extent of tonsillar herniation, hypothesized mechanisms, and associated neurologic symptoms vary. The most common cause of CMI is cranial constriction resulting from an underdeveloped posterior fossa (PF); other proposed mechanisms include cranial settling, spinal cord tethering, intracranial hypertension, and intraspinal hypotension [8]. The mechanism of cranial settling and joint instability may explain the co-occurrence of connective tissue disorders in some patients with CMI [9]. Symptoms of CMI vary widely in severity and often include headache, dizziness, neck pain, fatigue and difficulty swallowing [10].

Several lines of evidence exist that support a genetic contribution to CMI. These include twin studies, familial clustering, and co-segregation with known genetic syndromes (reviewed in [11]). However, little is known about the underlying genetic factors, and the clinical heterogeneity of CMI suggests that it is also genetically heterogeneous. A case–control candidate gene association study identified four single nucleotide polymorphisms (SNPs) in the caudal type homeobox 1(*CDX1*), fms-related tyrosine kinase 1 (*FLT1*), and aldehyde dehydrogenase 1 A2 (*ALDH1A2*) genes that were significantly associated (FDR < 0.10) with CMI when the study population was restricted to 186 patients. These patients were determined to have a small PF by MRI measurement [12]. A whole genome screen conducted in 2006 reported evidence for linkage to regions on chromosomes 9 and 15 using 23 non-syndromic CMI multiplex families [13]. Our group has carried out two additional whole genome screens. In the first screen, we used 66 non-syndromic CMI multiplex families and conducted a stratified linkage analysis using clinical criteria to reduce the genetic heterogeneity [11]. Specifically, families were stratified based on presence of hereditary connective tissue disorders. This approach resulted in a marked increase in evidence for linkage to multiple regions of the genome. In particular, those families without presence of connective tissue disorders showed regions of linkage in chromosomes 8 and 12, both of which contain growth differentiation factors (GDF3 and GDF6, respectively). In the second genome screen, an ordered subset analysis (OSA) using heritable and disease-relevant cranial morphologic traits identified increased evidence for linkage within subsets of families with similar cranial morphology. Results from OSA identified multiple genomic regions showing increased evidence for linkage, including regions on chromosomes 1 and 22 which implicated several biological candidates for disease [14].

Dura mater tissue surrounds the brain and spinal cord and is the final layer of the meninges, being located between the pia-arachnoid and bone. It is also a connective tissue, which is important because of the previously observed co-occurrence of CMI and connective tissue disorders [9,11,15]. Therefore, dura is a reasonable candidate tissue to examine in order to better understand the genetic causes of CMI.

Another study that used the same patient cohort as the present article identified CMI subtypes based on a clustering analysis of blood gene expression, dura gene expression, and cranial morphometrics [16]. These subtypes helped explain the clinical heterogeneity of CMI and implicated biological candidates responsible for this heterogeneity. Nonetheless, there was generally little concordance observed between the blood and dura mater gene expression profiles. Importantly, this study did not incorporate the patients’ genotypes in the analysis.

The goals of the present study were three-fold: 1. To illustrate the relative advantages of a joint-tissue approach to eQTL analysis, 2. To assess the concordance of eQTLs in blood and dura tissue, and 3. To explore the potential relevance of the identified eQTLs to CMI pathogenesis. All study participants underwent decompression surgery of the skull and dura samples were obtained during surgery. However, because dura tissue is much less accessible than blood, studies have preferentially analyzed eQTLs in blood for a range of clinical phenotypes, leaving dura expression under studied and also not represented in the GTEx project. By collecting both dura and blood expression levels for a common cohort we may determine where it is appropriate to use blood as a proxy for dura expression. Moreover, the integration of genotype and expression data through eQTL analysis provides novel information about potential candidate genes involved in the etiology of CMI.

## Results

### Tissue-by-tissue eQTL analysis

**Figure 1:**
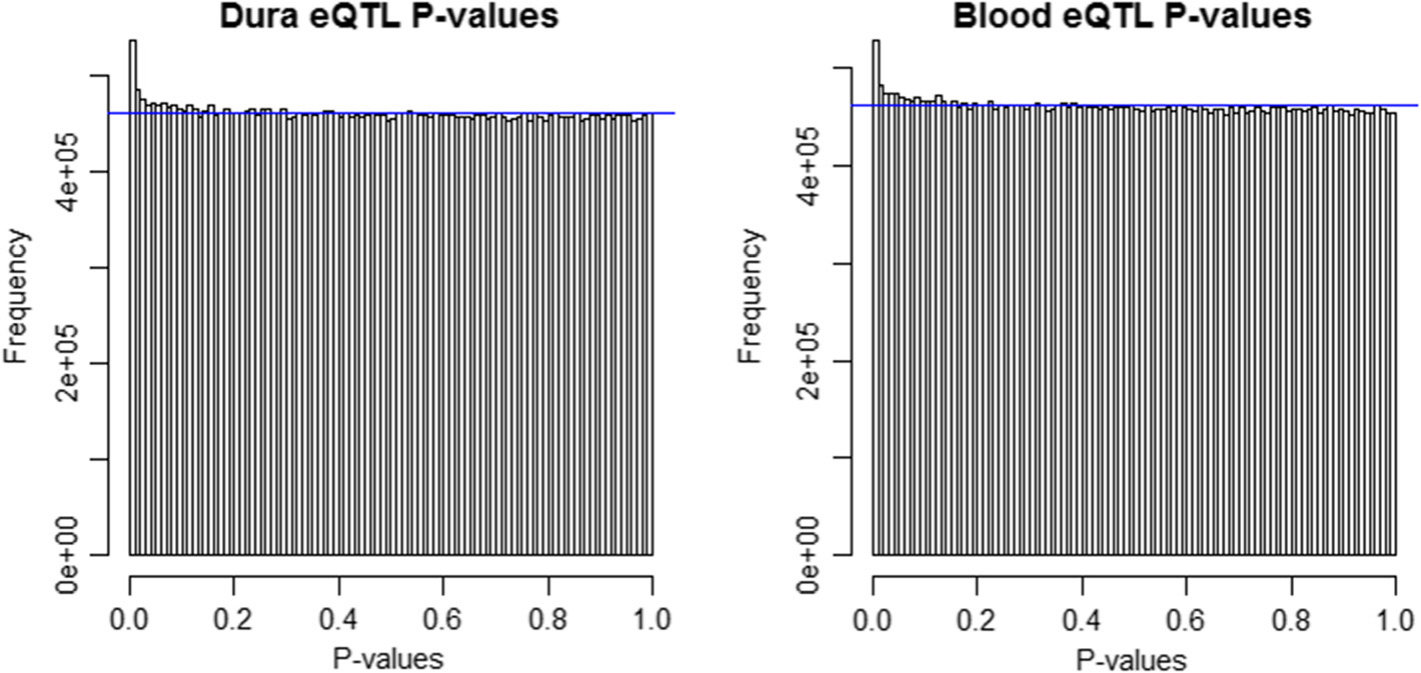
**Cis-eQTL p-value histograms.** Histogram of cis-eQTL p-values using a 1 Mb cis-region for dura (left) and blood (right) tissue. The horizontal blue line corresponds to a uniform distribution of p-values.

**Figure 2:**
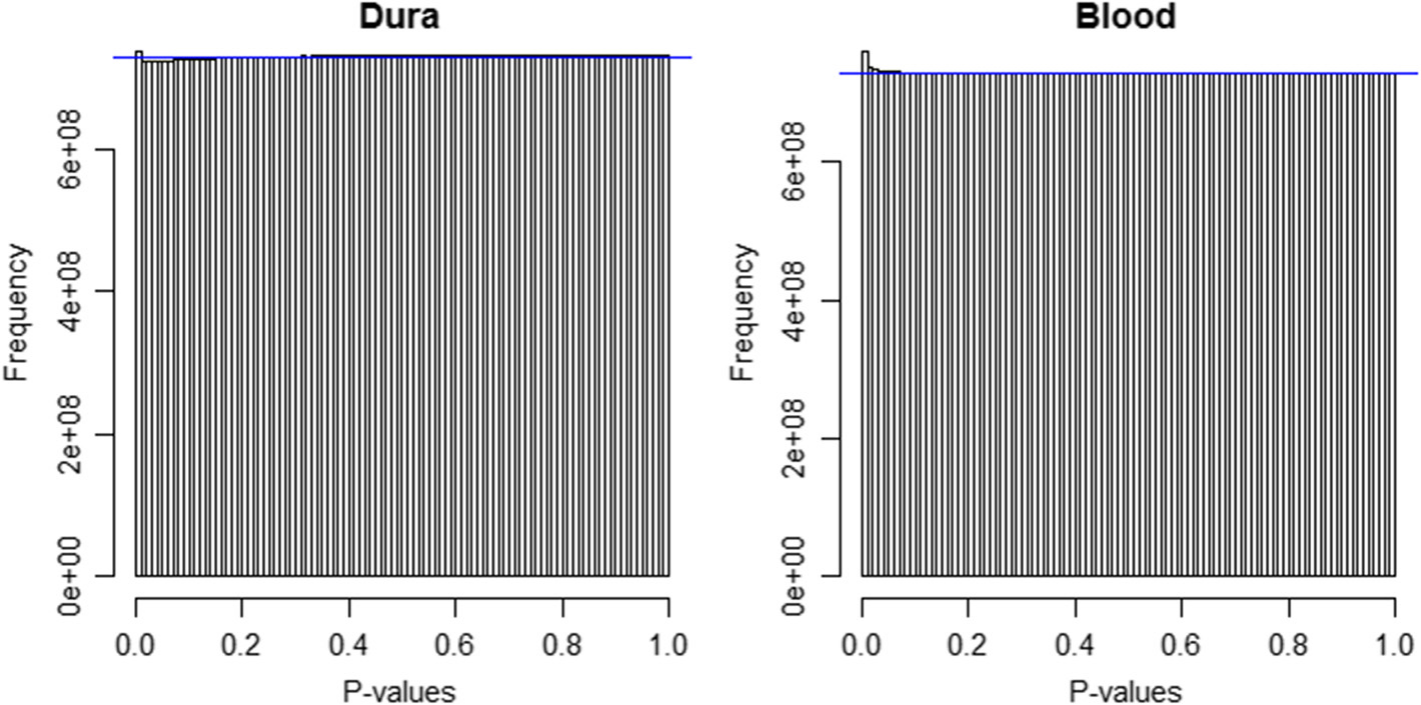
**Trans-eQTL p-value histograms.** Histogram of trans-eQTL p-values for dura (left) and blood (right) tissue. The horizontal blue line corresponds to a uniform distribution of p-values.

Separate eQTL analyses for blood and dura tissue were performed as described in the Methods section. The analyses included data for 43 individuals, with expression for 18,557 genes and genotype for 3,926,229 SNPs. A distinction was made between local (cis) and distant (trans) gene-SNP pairs. A histogram of p-values for significance of each cis gene-SNP association is shown in Figure 1, separately for dura and blood. A histogram of p-values for significance of each trans gene-SNP association is shown in Figure 2, separately for dura and blood. All plots are relatively uniform with a marked increase in frequency near 0. This indicates that some gene-SNP pairs have a highly significant association, but the vast majority of the pairs have no detectable association. The cis analyses have a much more pronounced peak near 0, indicating that for both tissues local eQTLs are substantially more likely to be active than trans-eQTLs, as expected.

There were 81 genes with a highly significant cis-eQTL in blood, and 175 genes with a highly significant cis-eQTL in dura tissue (FDR < 0.01); of these, 34 genes were significant in both blood and dura tissues. There were 163 genes with a highly significant trans-eQTL in blood, and 187 genes with a highly significant trans-eQTL in dura tissue (FDR < 0.01); of these, 12 genes were significant in both blood and dura tissues. These data are summarized in Table 1. Fisher’s exact test for association was highly significant for both the cis and trans tables (p-value < 0.001), suggesting that the overlap in eQTLs between the two tissues is not due to chance. Data for all trans-eQTLs are provided in Additional file 1.

**Table 1:** **Gene eQTL two-way tables (separate blood vs. dura analyses)**

	**Cis analysis**		**Trans analysis**	
**Dura**	**FDR < 0.01**	**FDR > 0.01**	**FDR < 0.01**	**FDR > 0.01**
**Blood**				
**FDR < 0.01**	34	141	12	175
**FDR > 0.01**	47	18335	151	18243

**Figure 3:**
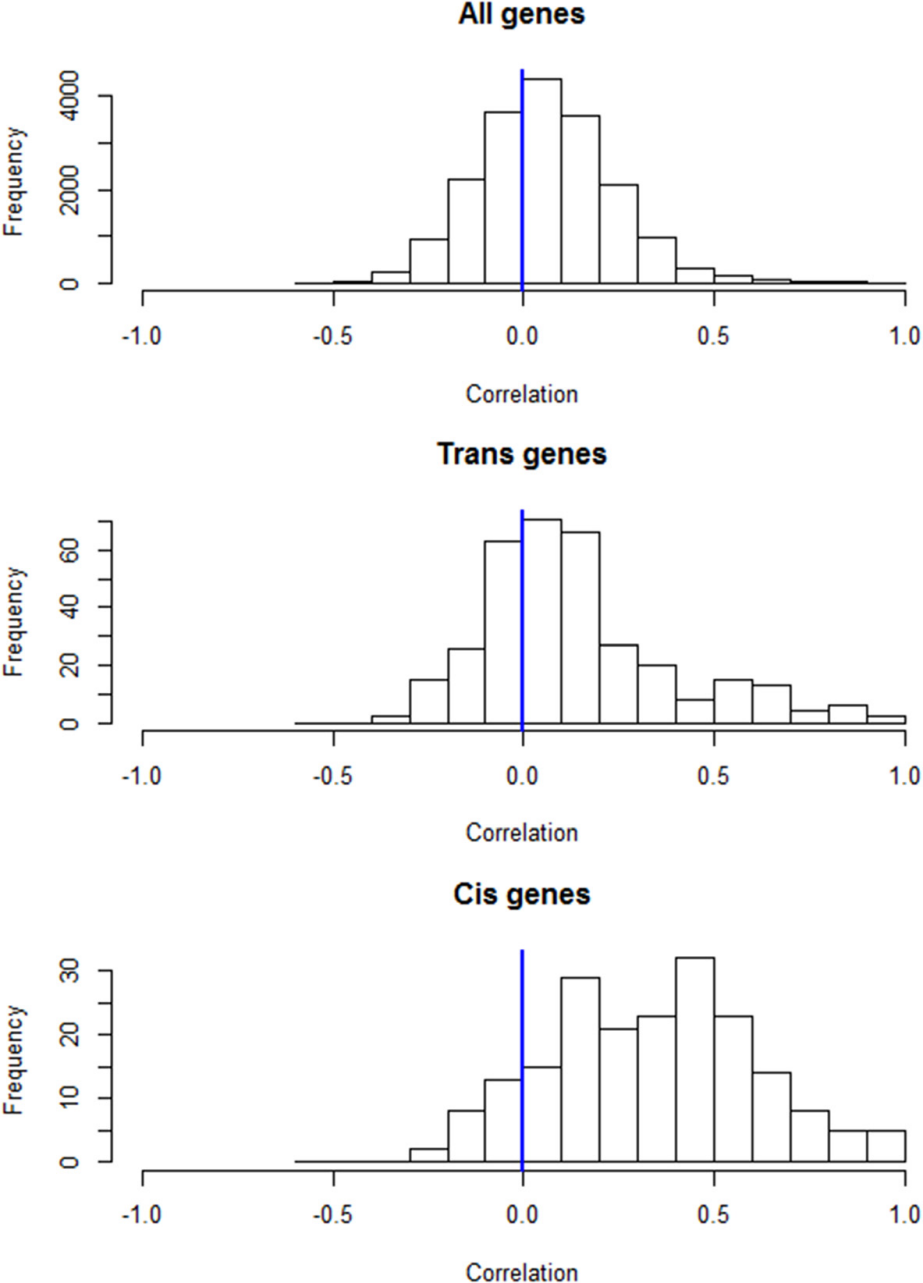
**Histogram of correlations for dura vs. blood expression.** Pearson correlations between dura and blood expression values are shown for all genes (top), those genes that had a trans-eQTL with FDR q < 0.01 in either tissue (middle), and those genes that had a cis-eQTL with FDR q < 0.01 in either tissue (bottom).

For cis-eQTLs the partial variability in expression that is explained by the given SNP (R^2^) must be greater than 45.2% to satisfy FDR < 0.01 for dura and 48.1% to satisfy FDR < 0.01 for blood. Thus, using stringent thresholds we only identify those eQTLs with a large effect. Further inspection suggested that the number of cis-eQTLs shared by both tissues was drastically underestimated by considering the intersection of separate analyses. Figure 3 shows the distribution of correlations between blood and dura expression for all 18,557 genes considered, for the 314 genes with significant trans-eQTLs only, and for the 198 genes with significant cis-eQTLs only. The distribution of correlations for all genes is centered near 0 and nearly symmetric, the distribution for trans-genes is centered near 0 with a slight right skew, the distribution for cis genes is shifted dramatically to the right and 88% of cis genes have a positive correlation. Hence, the vast majority of genes show no detectable association of expression between tissues, with the exception of genes with a cis-eQTL. This suggests that most cis-eQTLs are shared between the two tissues, and that the separate eQTL analyses are underpowered. These results motivated the joint-tissue analysis described below.

### Joint-tissue eQTL analysis

**Figure 4:**
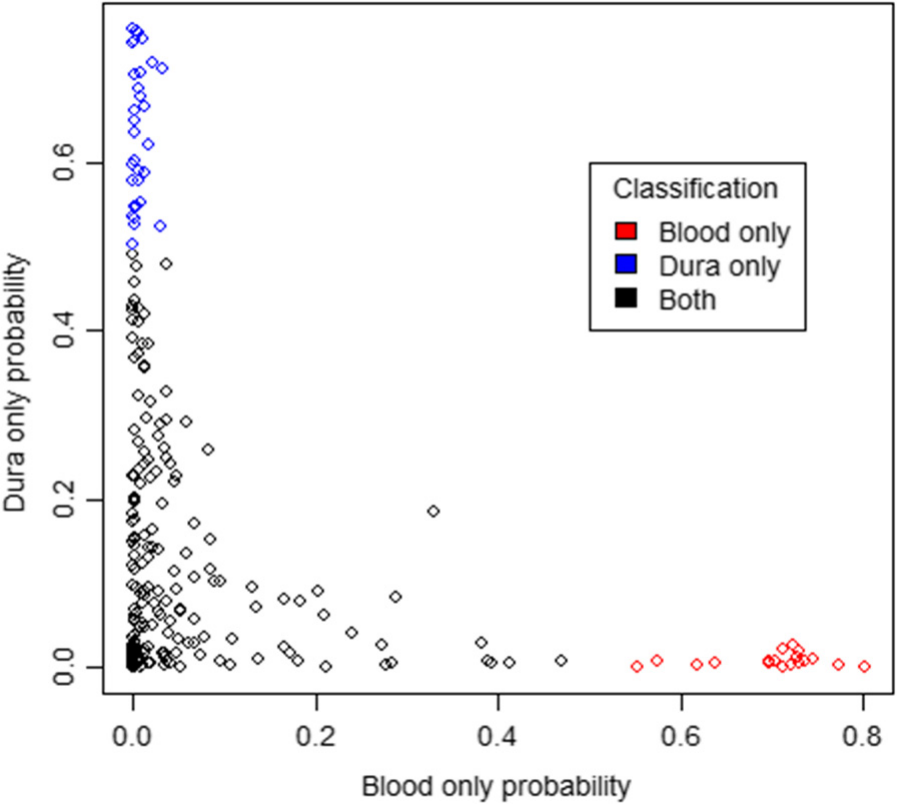
**Probabilities for eQTL tissue specificity.** Scatterplot of posterior probabilities that an eQTL is present in blood only or dura tissue only. The strongest eQTL for each gene with an FDR q < 0.01 under the joint analysis are shown. Each eQTL is colored by its posterior prediction for tissue activity (blood only, dura only, or both tissues).

Joint analysis of blood and dura tissue allowed for more accurate assessment of which eQTLs are active in blood only, dura only, or both tissues. Permutation testing under the joint model identified 239 genes with a highly significant (FDR < 0.01) cis-eQTL in either tissue. The majority (64%) of these genes were also identified by at least one of the tissue-specific analyses. For these 239 genes we focused on model comparison using Bayesian posterior estimates to determine if that gene had an active eQTL in blood only, dura only, or both tissues. Posterior probabilities that each gene had an active eQTL in blood only or dura only, as determined by the most highly associated SNP, are shown in Figure 4. Of these eQTLs, 18 had a higher probability of being active in blood only (relative to “dura only” or “both”), 33 had a higher probability of being active in dura only, and 188 were predicted to be active in both tissues. A contingency table showing the agreement in gene classification between the joint-tissue and separate analyses is given in Table 2. For a more direct comparison, we also performed gene-level permutation-testing with the same software used for the multi-tissue analysis but with no inter-tissue dependence; this gave 126 cis-eQTLs in dura and 77 cis-eQTLs in blood (FDR < 0.01), with 32 shared by both tissues. For both comparisons many more genes were predicted to have jointly present eQTLs using the multi-tissue analysis than by simply considering the intersection of separate analyses. This result was expected, given the high level of between tissue correlation for genes with eQTLs.

**Table 2:** **Gene eQTL contingency table (separate vs. joint analyses)**

		**Joint**		
**Separate**	**Blood only**	**Dura only**	**Both tissues**	**Neither, FDR > 0.01**
**Blood only**	12	0	0	6
**Dura only**	0	28	0	5
**Both tissues**	14	66	33	75
**Neither, FDR > 0.01**	21	47	1	18249

Additional file 2 gives results from the multi-tissue analysis for all genes considered. Additional file 3 lists each of the 239 significant genes with additional information for each gene. We compared the strongest eQTL for each gene with association p-values from the gTEX database for blood. As expected, most (76%) of eQTLs that were identified as active in blood only also had a gTEX p-value < 0.05 and less (24%) of eQTLs that were identified as active in dura only were significant in the gTEX database. Interestingly, only 37% of eQTLs that were determined to be active in both blood and dura had a p-value < 0.05 in the gTEX database.

**Figure 5:**
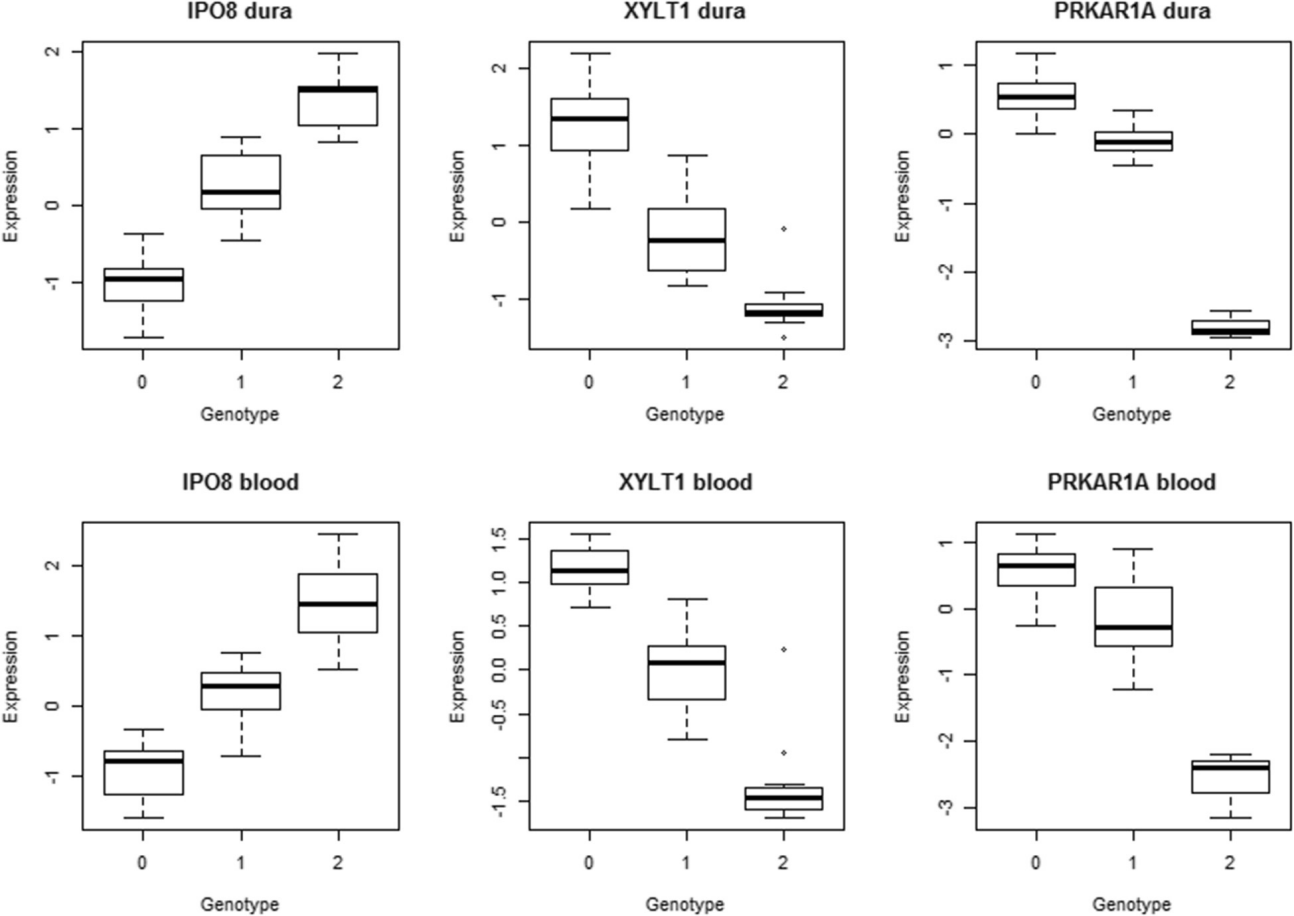
**Expression vs. genotype boxplots.** Boxplots show the association between IPO8 expression and the SNP rs10743724, between XYLT1 expression and the SNP rs1045885, and between PRKAR1A expression and the SNP rs2302234, for dura (top) and blood (bottom) tissues. Expression values are z-standardized after preprocessing. The genotype is coded by number of copies of the minor allele (0, 1, or 2). All plots show a clear trend.

Among all genes considered, 6.6% had a measured SNP in at least one of its probe targets. Among the 239 genes identified, 36 (15.1%) had a SNP in a probe, and in 12 such genes (5.0%) the eQTL SNP was in LD (${R}^2>0.5$) with at least one SNP in a probe for that gene. These eQTLs were flagged as potential artifacts due to hybridization, and this information is given in Additional file 3. We prioritized those genes that had eQTLs active in dura only, or that were strongly active in both tissues, but not significant in the gTEX database, because we deemed these eQTLs more likely to be relevant to CMI pathophysiology. Among these prioritized eQTLs, the genes importin 8 *(IPO8*), xylosyltransferase I (*XYLT1*), and protein kinase cAMP-dependent regulatory type I alpha (*PRKAR1A*) were all found to have a very strong eQTL in both blood and dura tissue. These eQTLs had an FDR < 0.001 in the joint and tissue-by-tissue analyses, and the SNP-gene association showed a strong linear trend in both tissues (Figure 5). All three eQTLs did not show a significant association in the GTEx database. However, IPO8 expression was associated with a SNP in its probe target. We elaborate on the biological function and potential relevance to CMI for each of these genes in the Discussion.

### Family-based association test (FBAT)

A FBAT was performed on an independent familial population to assess the presence of genotype associations with the occurrence of CMI. Neither a full FBAT including over 4 million SNPs, or a reduced FBAT analysis restricted to those 239 SNPs identified by the joint-tissue eQTL analysis above, resulted in any highly significant associations after an FDR adjustment (FDR < 0.05). Nevertheless, p-values resulting from the FBAT analysis for 239 SNPs are listed in Additional file 3, and provide additional support for certain candidate genes. In particular, *ribosomal protein 23* (RPS23) had a strong eQTL in both tissues (FDR < 0.001), no significant eQTL in the GTEx database, and a small FBAT nominal p-value (p-value = 0.01). Together these data suggested a potential role of RPS23 in the development of CMI.

Furthermore, we considered those eQTLs within a linkage region for CMI, as identified by the previous stratified linkage [11] and ordered subset [14] analyses. Of all SNPs considered, 3.0% belong to such a region. Of the 239 eQTLs identified by the joint tissue analysis, 10 belonged to such a region and these are annotated in Additional file 3. The enrichment of SNPs belonging to such a region among these 239, relative to all SNPs, was not significant (P-value = 0.18; Fisher’s exact test).

### Network and pathway analyses

**Figure 6:**
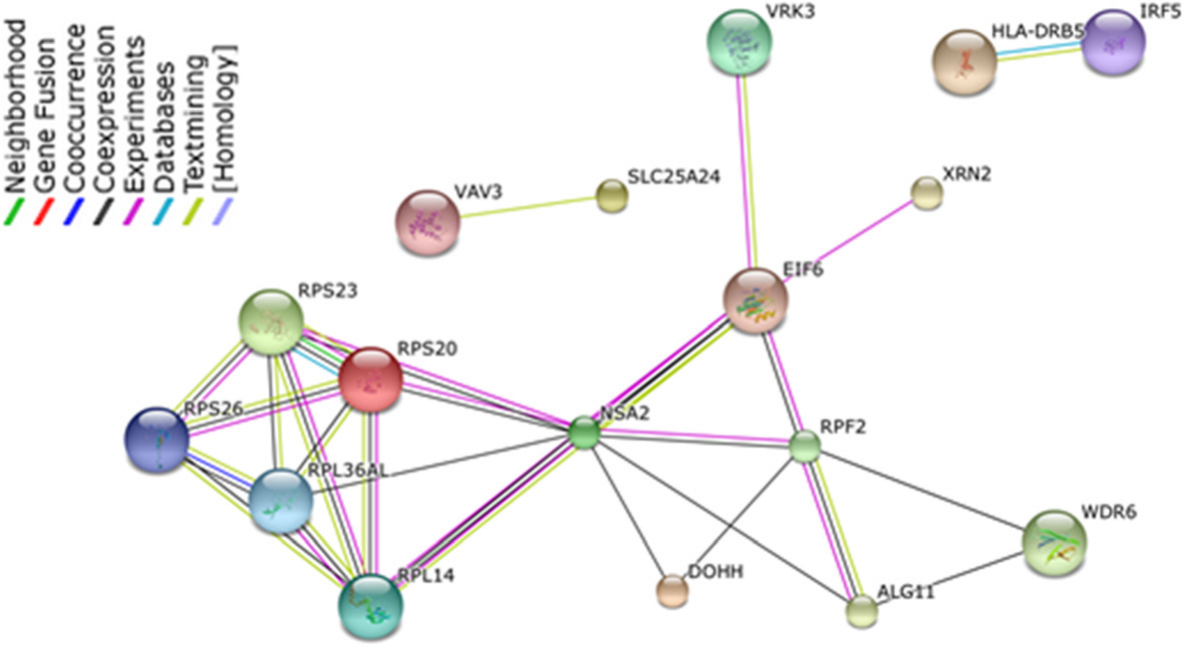
**Significant gene network.** Network of genes with associated functional protein interactions, created based on genes with strong eQTLs in blood and dura (log Bayes factor > 10) or significant eQTLs in dura only. This included 64 genes, 47 of which were isolated as they had no functional interactions with the other genes; the remaining 17 genes are shown.

A functional protein interaction network based on the joint-tissue eQTL analysis is shown in Figure 6. For relevance to CMI, only those genes with a strong eQTL in both dura and blood (log Bayes factor > 10) or a significant eQTL in dura only were included in the network (n = 64 genes). The majority of genes do not interact with one another based on interactions within STRING (data not shown). However, a common network connects several genes associated with ribosome function. These genes include the ribosomal proteins RPS26, RPS23, RPS20, RPL14, RPL36AL, the ribosome biogenesis homolog NSA2, and the ribosome production factor homolog RPF2. Of the 14 genes that are included in this network, 8 have their strongest eQTL predicted to occur in dura only (RPS20, RPL36AL, NSA2, EIF6, VRK3, RPF2, DOHH, and WDR6). Of the remaining 5 genes, 4 did not have their strongest eQTL replicated in the gTEX database (p-value > 0.1) (RPS23, RPL14, XRN2, ALG11); only RPS26 occurred in both dura and blood and shared its most significant eQTL with the gTEX database. A pathway enrichment analysis of genes predicted to have eQTLs in blood and dura or dura only (n = 221 genes) was performed using both GO and KEGG pathways. The only pathway that was significantly enriched in this gene set at FDR < 0.05 was the KEGG *ribosome* pathway, including genes RPS20, RPL36AL, RPL22L1, RPS26, RPS23, RPL14.

Together, these results indicate that coordinated activity among multiple genes from the joint-tissue eQTL analysis is primarily related to ribosomal function. The eQTL associated with most of these genes occurs in dura only, or is not significant in the gTEX database, suggesting that these eQTLs may be related to CMI.

## Discussion

A comparison of the tissue-by-tissue and joint-tissue analyses illustrates the advantages of an integrative multi-tissue approach to eQTL analysis. The joint-tissue analysis identified more significant eQTLs overall, suggesting that borrowing information across tissue types increased statistical power. In particular, the combined use of exploratory plots and the joint-tissue analysis suggested that the number of eQTLs shared by both blood and dura was vastly underestimated when simply taking the intersection of tissue-by-tissue analyses. Consequently, the number of tissue-specific eQTLs was overestimated. The joint-tissue analysis gives a more principled way to assess whether an eQTL is shared or specific to a given tissue.

We found substantially higher between-tissue correlation in genes with cis-eQTLs than genes with trans-eQTLs; this agrees with studies of eQTL specificity on other tissues [17,18]. Approximately 79% of cis-eQTLs that were detectable in either dura or blood were predicted to be shared by both tissues. The only previous comparison of expression levels for blood and dura, using the same sample set, found little concordance between the two tissues [16]; however, this analysis did not investigate eQTLs. We similarly found that the association between expression in blood and dura tissue was generally negligible, but importantly, we found high levels of association in expression of genes with cis-eQTLs. This is almost certainly because most cis-eQTLs are shared across the tissues. We posit that genes with eQTLs are more likely to play an important role in conditions with a genetic component, such as CMI. Thus, our conclusions provide some support for the utility of future biomarker development for CMI in less invasive tissues, such as blood.

We identified three genes with strong eQTLs in both blood and dura tissues that were not significant in the gTEX database and had function that was potentially relevant to CMI: IPO8, XYLT1, and PRKAR1A. IPO8 expression was previously associated with osteoblast differentiation [19], and found to be increased 25-fold in patients with Os odontoideum in a comparative twin study [20]. These results suggest that IPO8 may play a role in bone development, a process believed to underlie the etiology of CMI in at least some cases [12,21]. XYLT1 activity has also been implicated in ossification [22-24], specifically as a regulator of chondrocyte maturation [22,24]. Mutations in PRKAR1A have been associated with Acrodysostosis, a genetic disorder of bone growth [25]. These candidate genes all relate to bone growth and development, and their relevance to CMI is supported by evidence that cranial morphometrics are heritable within CMI families and associated with CMI case status [12,16].

Several genes with strong eQTLs were related to ribosomal function. These genes had eQTLs primarily in dura tissue only, or had an eQTL in both blood and dura tissue, but were not significant in the gTEX database. This suggests that ribosomal expression may be relevant to the etiology of CMI. Mutations and expression of genes encoding ribosomal proteins have been implicated in a variety of conditions related to bone marrow dysfunction and disorders of skeletal development. These include the bone marrow disorders Diamond-Blackfan anemia and Schwachman-Diamond syndrome, and the skeletal development disorders Cartilage-hair hypoplasia and Treacher Collins syndrome [26,27]. Treacher Collins syndrome is characterized by craniofacial abnormalities [28]. There is also a link between ribosomal bone marrow disorders and skeletal conditions, as both Diamond-Blackfan anemia and Schwachman-Diamond syndrome have been associated with craniofacial and skeletal anomalies [29,30]. Several cases have been reported where CMI and bone marrow disorders co-occur [31,32]. However, the causal association between ribosomal function and CMI, if any, requires further examination. Our study participants were examined for the presence of anemia based on a review of medical records, and only two patients were identified with low mean corpuscular volume (MCV) values (data not shown). It remains unclear if the ribosomal protein associations observed in the present study are representative of a link between CMI and underlying anemia.

Gene expression of dura tissue has not been well studied, in part because of its inaccessibility without invasive surgery. While the present study allowed a unique opportunity to examine dura expression, its scope of inference is limited to young patients with CMI. Dura tissue is a natural candidate for the study of CMI because of its proximity to cranial bone, and the co-occurrence of CMI and connective tissue disorders [11]. However, there may be important genetic factors in the development of CMI that are not manifested in blood or dura tissue, but are best characterized within the bone itself, or other tissue types.

While the joint–tissue analysis improves power, it is still limited by the study sample size and conservative criteria for significance. Only eQTLs with strong relative effects on gene expression are identified, and so these results do not represent a comprehensive catalogue of eQTLs in blood and dura tissues.

The interpretation of eQTL results requires caution, as not all observed gene-SNP associations may be caused by true regulatory effects. Some associations may be due to technical artifacts such as RNA hybridization issues, and we have attempted to flag suspect associations. Furthermore, because the present study was restricted to patients with CMI it is subject to the effects of conditioning on a “collider” [33]. Specifically, an association may be observed in a gene-SNP pair if both the gene and the SNP are independently associated with CMI status, even if there is no true regulatory effect.

## Conclusions

We have presented the first joint-eQTL analysis of dura mater and blood. We demonstrated that the integrative statistical approach of joint-eQTL analysis is more powerful than identifying the intersection of single tissue analysis. Our significant eQTLs revealed functionally relevant and novel candidate genes for the pathology of CMI and provide the basis of further exploration.

## Methods

### Ethics statement

The details of this study were approved by the Duke University Medical Center Institutional Review Board (protocol 00020342).

### Study population

Eligible study participants were pediatric patients diagnosed with CMI and treated with PF decompression surgery at Duke University Medical Center over a period of 20 months. All participants were under 18 years old but were otherwise of varied age, sex and race. Table 3 gives detailed population characteristics and available data for the 44 participants. Participation required written consent for the release of medical records, providing a blood sample for DNA and RNA extraction, and providing a dura sample for RNA extraction. Choice to participate in the study did not affect the patient's quality of care, and only 7.9% of those eligible declined enrollment.

**Table 3:** **Study population description**

**Description**	**N**	**Percentage**
**Total number of individuals**	44	
**Sex**		
Male	28	63.6%
Female	16	36.4%
**Race**		
Caucasian	31	70.5%
African American	13	29.6%
**Syrinx**		
Yes	10	22.7%
No	34	77.3%
**Family history**		
Yes	6	13.6%
No	36	81.8%
Unknown	2	4.6%
**Datasets**		
Blood gene expression	44	100.0%
Dura gene expression	44	100.0%
Genotype	43	97.7%
**Age at surgery (years)** ^**a**^	8.89 ± 5.19	

^a^Average age at surgery ± standard deviation.

### Laboratory protocols

#### Sample collection and storage

Samples of dura mater and blood tissue were collected from all participants during PF decompression surgery. Decompression surgery involves removing a small portion of the back of the skull to alleviate pressure near the brainstem and spinal cord, and was performed under anesthesia. All patients underwent duraplasty, in which the open dura mater was closed with a cadaveric pericardial patch graft. During this procedure, a small piece of dura tissue (<5 mm × 5 mm) was collected and stored in a tube filled with 1.25 ml of RNALater (Life technologies, Grand Island, NY). Also during surgery, blood samples were collected in a 2.5 ml Paxgene RNA tube (Qiagen, Valencia, CA) from an arterial line. For DNA extraction, blood samples were collected from study participants in EDTA tubes.

#### RNA extraction and expression arrays

RNA was extracted within one month after ascertainment for each blood and dura sample. Prior to RNA extraction for dura, the samples were homogenized at 4°C in 2 ml Omni bead ruptor tubes prefilled with 2.38 mm metal beads and buffer RLT (Qiagen, Valencia, CA) plus β-Mercaptoethanol. RNA extraction, DNAse treatment, and clean-up for dura were performed using the RNeasy fibrous tissue mini kit (Qiagen, Valencia, CA), according to the manufacturer's protocol. RNA extraction and DNAse treatment for blood were performed using the PAXgene Blood RNA kit (Qiagen, Valencia, CA), according the manufacturer's protocol.

Nanodrop (ThermoScientific, Wilmington, DE) was used to quantify the RNA for both blood and dura tissue. All samples had a total yield of at least 50 ng. The RNA 6000 Pico chip (Agilent, Santa Clara, CA) was used to assess the RNA Integrity Number (RIN). All samples had a RIN score over 6, indicating the RNA was of satisfactory quality.

Prior to RNA amplification, samples were concentrated using a vacuum centrifuge. The TotalPrep-96 RNA Amplification Kit (Illumina, San Diego, CA) was then used to amplify and convert the RNA samples to cRNA per the manufacturer's instructions. All 44 dura samples, 44 blood samples, a positive control included in the kit, a dura control sample (Clontech human dura matter total RNA), and a blood control sample (Clontech human blood, peripheral leukocytes total RNA) were all run on the same 96-well plate. The cRNA samples were then diluted to a concentration of 150 ng/ul.

Whole genome expression data was generated for all samples using the HT-12 v4 Expression BeadChips (Illumina, San Diego, CA) per the manufacturer’s instructions. All samples were run in a single experimental batch. The dura samples were distributed across 4 chips, with the dura control sample run on each chip; the blood samples were distributed across 4 other chips, with the blood control sample run on each chip. The age, race, gender, and operating surgeon for the 44 samples were approximately evenly distributed between the 4 chips.

#### DNA extraction and genotyping arrays

DNA was extracted from EDTA tubes using the AutoPure LS® DNA extraction kit with Puregene® system reagents (Qiagen, Valencia, CA). A small amount of DNA (0.3 μg) was run on a 0.8% agarose gel in order to assess quality and each sample was quantified using the Nanodrop (ThermoScientific, Wilmington, DE). Whole-genome genotype data was generated via the Illumina Human610-Quad BeadChip (Illumina, San Diego, CA) per the manufacturer’s instructions and chips were scanned using the Illumina iScan system (San Diego, CA). Genotyping for the 44 participants described in this study was performed in a single batch.

### Data processing

#### Whole genome expression quality control and data pre-processing

The GenomeStudio Gene Expression module (Illumina, San Diego, CA) was used for initial quality assessment of the blood and dura expression data. System controls were checked and found to in agreement with expected performance. Blood and dura control replicates were assessed for consistency. There was high concordance between replicates, as the Pearson correlation coefficient was > 0.99 for dura and > 0.98 for blood.

Sample outliers were identified based on the number of genes with low detection p-values, signal intensity measures, housekeeping gene intensity, and other system control metrics. The distribution for each metric was measured separately for blood and dura, and a sample was flagged as an outlier if it was more than 4 standard deviations from the mean in any metric. Of the 15 sample-level metrics used, one dura sample was flagged based on two metrics: signal average and housekeeping gene intensity. No blood samples were identified as outliers in any metric. No blood or dura samples were removed from analysis based on these metrics.

After initial quality assessment, the R environment [34] was used for pre-processing of expression data. Probe intensities were log2-transformed then quantile normalized using the R package lumi [35]. All control replicates clustered together based on hierarchical clustering in lumi. Principal components analysis (PCA) was performed separately for blood and dura samples to identify outliers and clustering anomalies. No samples were flagged as outliers, and we concluded that clustering present in PCA plots represents real biological variation. Sex of the samples was confirmed by examining expression of probes on the Y chromosome, and all samples grouped with other samples of their reported sex.

The 47,210 probes mapped to 18,557 RefSeq genes, and the mean of the normalized probe expression values was used to summarize expression for each gene. Pearson correlation between normalized probe expression was used to assess the agreement of probes that map to the same gene.

#### Genotyping quality control and imputation

Genotype calls were made using GenomeStudio (Illumina, San Diego, CA) and quality control (QC) was conducted in PLINK [36]. QC was performed separately for the Caucasian (N = 31) and African-American (N = 13) subjects due to varying minor allele frequency (MAF) and linkage disequilibrium (LD) across ethnic groups. All samples had call rates > 99%. X chromosome heterozygosity was assessed and one Caucasian sample was excluded due to discrepancy between genotype and clinical definitions of gender, leaving N = 43 eligible samples. Identity-by-descent (IBD) estimates were calculated to identify duplicate or related individuals and principal component analysis (PCA) was used to check for population substructure; no subjects were removed for these reasons. SNPs with MAF < 5% or Hardy-Weinberg Equilibrium (HWE) p-values < 0.001 were removed. After initial QC, 518,054 SNPs remained for the Caucasian subjects and 489,095 SNPs remained for the African-American subjects.

To increase genome-wide coverage, we imputed missing genotypes using the 1000Genomes (www.1000genomes.org) global reference panel. Samples were first phased using SHAPEIT [37] and genotypes were subsequently imputed using IMPUTE2 [38]. Imputed probes with certainty values < 90% were zeroed out and SNPs missing in more than one person, corresponding to a SNP call rate of 92.3% for the Caucasian subjects and 96.7% for the African-American subjects, were removed. SNPs with MAF < 5% across both populations were also removed. Accuracy of the imputed data was assessed by masking SNPs genotyped on the Illumina panel and comparing the imputed genotypes to the observed genotypes. The overall concordance was 98.7%. After all quality control steps, 3,926,229 SNPs remained.

### Data analysis

#### Tissue-by-tissue eQTL analysis

Independent eQTL analyses for blood and dura tissue were performed using *Matrix EQTL* [2]. The strength of association between a given gene-SNP pair was measured using an additive linear model. The variables sex, race and age were included as covariates for adjustment. For a given gene-SNP pair the full model was

$$ Expressio{n}_j={\beta}_0+{\beta}_{snp}\cdot SN{P}_j+{\beta}_{sex}\cdot SE{X}_j+{\beta}_{race}\cdot RAC{E}_j+{\beta}_{age}\cdot AG{E}_j+{\epsilon}_j, $$

where

- *Expression*_*j*_ is log-normalized expression for the given gene, for sample *j*.
- *SNP*_*j*_ is the minor allele count (0,1,2) for the given SNP, for sample *j*.
- *SEX*_*j*_ is the sex of sample *j* (0 = female, 1 = male)
- *RACE*_*j*_ is the race of sample *j* (0 = Caucasian, 1 = African American)
- *AGE*_*j*_ is the age, in years, of sample *j.*

Significance testing for the hypothesis of no SNP-gene association (*β*_*snp*_ = 0) was performed assuming independent Gaussian errors *∈*_*j*_

We tested the association of all gene-SNP pairs (18,557 genes × 3,926,229 SNPs), for blood and dura tissue. A distinction was made between local (cis) and distant (trans) regulatory associations. Specifically, gene-SNP pairs in which the given SNP was within 1 Mb upstream or downstream of the RefSeq coding region for the given gene were considered cis, while all other pairs were considered trans. To improve power when adjusting for multiple comparisons, the Benjamini-Hochberg false discovery rate (FDR) [39] was computed separately for cis- and trans-eQTLs.

#### Joint-tissue eQTL analysis

Joint eQTL analysis of blood and dura tissue was performed using the *eQTL Bayesian Model Averaging* (eqtlbma) method [5]. As with the separate analyses, eQTLs were identified using an additive linear model with sex, race and age included as covariates for adjustment; a model for dependence between tissues was also used for the association of each gene-SNP pair. For a given gene-SNP pair and tissue *s* (*s =* blood or *s* = dura), the full model was

$$ Expressio{n}_{j,s}={\beta}_{0,s}+{\gamma}_s{\beta}_{snp,s}\kern0.5em SN{P}_j+{\beta}_{sex}\cdot SE{X}_j+{\beta}_{race}\kern0.5em RAC{E}_j+{\beta}_{age}\cdot AG{E}_j+{\epsilon}_{j,s}., $$

Where *Expression, SNP, SEX, and AGE* are defined as above, and γ_s_ defines whether an eQTL is active in tissue *s* (0 = inactive, 1 = active). A hierarchical normal random-effects model was used to account for dependence between the tissue-specific eQTL effects *β*_*snp*,*s*_. A Bayesian framework allowed for computation of the posterior probability (the probability given the data) for each of the following scenarios:

- eQTL not present in either tissue (*γ*_*blood*_ = *γ*_*dura*_ = 0)
- eQTL present in blood but not dura (*γ*_*blood*_ = 1; *γ*_*dura*_ = 0)
- eQTL present in dura but not blood (*γ*_*blood*_ = 0; *γ*_*dura*_ = 1)
- eQTL present in both tissues (*γ*_*blood*_ = 1; *γ*_*dura*_ = 1)

We employed a permutation testing procedure to assess significance for the hypotheses that a given gene is not associated with any SNP in either tissue. For more details on the Bayesian probability framework and permutation procedure used, see the Methods section of the eqtlbma article [5]. For significance testing we use FDR to control for multiple comparisons.

The multi-tissue model was estimated for local eQTLs only, with a cis region of 1 Mb. The eqtlbma software was used with default settings; this included estimating the model for a pre-specified grid of hyperparameter values and averaging posterior estimates. We used 10,000 permutations to assess gene-level significance. For highly significant genes we considered the SNP that is most highly associated with that gene, as determined by the posterior probability that *γ*_*blood*_ = 1 or *γ*_*dura*_ = 1, for further analysis and interpretation.

The GTEx data portal (http://www.gtexportal.org, accessed 01/12/2014) includes eQTL p-values for association between expression levels and minor allele frequency for most genes and SNPs considered in this study, for a variety of tissue types. Where available, we collected GTEx p-values for blood tissue for those gene-SNP pairs that were highly significant in our multi-tissue analysis, to assess whether these associations are replicated in a larger population that is not affected with CMI. GTEx data were available for 87% of the gene-SNP pairs considered.

SNPs that are within the target region of a probe may affect RNA hybridization and lead to false-positive eQTL findings [40,41]. With this in mind, gene-SNP pairs in which the given SNP had high LD (*R*^2^ > 0.5) with a SNP in the probe target region for the given gene were considered potential artifacts.

#### Family-based association test

We also conducted a family-based association test (FBAT) [42] on an independent cohort of 421 related individuals to explore the relationship between certain eQTL SNPs and incidence of CMI. This cohort includes multi-generational pedigrees for 66 families, with 183 affected (diagnosed with CMI) and 192 unaffected individuals. Linkage studies involving a sample from this cohort, for the same families, have been described previously [11,14]. Genotype data were generated and imputed as described above.

We performed FBAT for linkage and association, using default settings, for all 4,493,641 SNPs included in this cohort. We performed separate FDR corrections for multiple comparisons, one for all SNPs and the other for those SNPs that were identified as significant by the multi-tissue eQTL analysis.

#### Network and pathway analyses

To identify coordinated activity between genes, protein network and pathway enrichment analyses were performed on those genes that had highly significant eQTLs in blood and dura, or dura only, in the multi-tissue analysis. STRING [43] was used to construct functional protein association networks. WebGestalt [44] was used to perform enrichment analysis of Gene Ontology (GO) and Kyoto Encyclopedia of Genes and Genomes (KEGG) pathways.

## Additional files

Additional file 1:
**Significant Trans eQTLs.** This .xls spreadsheet provides data for all significant trans eQTLs (FDR < 0.01), with the blood and dura analyses as separate tabs. The given gene, the given SNP, the p-value, and the t-statistic are listed for each eQTL. Additional file 2:
**Joint-tissue analysis statistics.** This .xls spreadsheet provides joint-tissue results for all genes considered. Statistics given for each gene include its p-value under permutation, mean log Bayes factor, most highly associated SNP, and log Bayes factors for the given gene-SNP pair. Additional file 3:
**Significant gene table.** This .xls spreadsheet provides additional information for those genes with significant eQTLs (FDR < 0.01) in blood or dura mater based on the joint-tissue analysis. Statistics given for each gene include the mean log Bayes factor; the most highly associated SNP; the probability that the given gene-SNP pair is an eQTL in blood only, dura only, or both tissues; the p-value of the given SNP under a family based association test for association with CMI; whether the given SNP falls in a pre-identified linkage region; the p-value of the given gene-SNP pair in the gTEX database for blood; the number of probes that map to the gene; the SNPs that fall within a probe region, and the maximum ${R}^2$ value between the most highly associated SNP and a SNP within a probe region. A second tab in this spreadsheet gives probe-level information for those genes with multiple probes. For each gene the mean correlation between probes for blood and dura expression are provided; for each probe the standard deviation for blood and dura expression, and the ${R}^2$ value measuring association with the eQTL SNP, are provided.

## Abbreviations

(eQTL): Expression Quantitative Trait Loci
(CMI): Chiari Type I Malformation
(SNP): Single Nucleotide Polymorphism
(GTEx): Genotype-Tissue Expression
(RIN): RNA Integrity number
(RT-qPCR): Real-time quantitative PCR
(CV): Coefficient of variation
(PCA): Principal components analysis
(GO): Gene Ontology
(KEGG): Kyoto Encyclopedia of Genes and Genomes
(LD): Linkage disequilibrium
